# Prevalence and factors associated with illicit drug and high-risk alcohol use among adolescents living in urban slums of Kampala, Uganda

**DOI:** 10.1186/s12889-024-19250-x

**Published:** 2024-06-26

**Authors:** Hellen Kalungi, Onesmus Kamacooko, Jane Frances Lunkuse, Joy Namutebi, Rose Naluwooza, Matt A. Price, Eugene Ruzagira, Yunia Mayanja

**Affiliations:** 1 Medical Research Council/Uganda, Virus Research Institute and London School of Hygiene and Tropical Medicine (MRC/UVRI & LSHTM) Uganda Research Unit, Plot 51-59 Nakiwogo Road, P.O. Box 49, Entebbe, Uganda; 2 London School of Hygiene and Tropical Medicine, Keppel Street, WC1E 7HT London, UK; 3 International AIDS Initiative (IAVI), 125 Broad St, 10004 New York, NY USA; 4 Department of Epidemiology and Biostatistics, University of California San Francisco, 550 16th Street, 94143 San Francisco, CA USA; 5 Child Health and Development Center, College of Health Science, Makerere University, Kampala, Uganda

**Keywords:** Adolescents, Illicit drug use, High-risk alcohol use, Urban slums, Kampala, Sub-saharan Africa

## Abstract

**Background:**

Illicit drug and high-risk alcohol use among adolescents leads to poor health outcomes. We enrolled adolescents from urban slums in Kampala, Uganda, to assess baseline prevalence and factors associated with illicit drug and high-risk alcohol consumption.

**Methods:**

We conducted a cross-sectional study using data collected in a cohort that enrolled 14-19-year-old male and female participants from 25 March 2019 to 30 March 2020. Data was collected on social demographics, sexual behavior, and reproductive health using interviewer-administered questionnaires. The main outcomes were illicit drug use and high-risk alcohol use. Data on alcohol use was collected using the Alcohol Use Disorder Identification Test (AUDIT); results were dichotomized. Factors associated with each outcome were analyzed using multivariable logistic regression.

**Results:**

We enrolled 490 participants (60.6% female) with a median age of 18 (IQR 17–18) years, 84.9% had less than secondary education, 48.4% had their sexual debut before 15 years, 47.1% reported paid sex in the past 3 months and 22.8% had a sexually transmitted infection (chlamydia, gonorrhea, and active syphilis) baseline characteristics associated with illicit drug use in the past 3 months were male gender (aOR 12.45; 95% CI 7.21–21.50) being married (aOR 2.26; 95%CI 1.03–4.94) 10 or more paying sexual partners (aOR 2.45; 95%CI 1.05–5.69) and high-risk alcohol use (aOR 3.94; 95%CI 2.10–7.40), baseline characteristics associated with high-risk alcohol use were male gender (aOR 0.29; 95% CI 0.13–0.63) emotional violence from sexual partners (aOR 2.35; 95%CI 1.32–418) illicit drug users com (aOR 3.94; 95% CI 2.10–7.40).

**Conclusion:**

Illicit drug and high-risk alcohol use are prevalent among male adolescents and adolescents involved in high-risk sexual behavior living in the urban slums of Kampala.

## Introduction

The 2021 United Nations Office on Drugs and Crime (UNODC) report, estimates that 275 million individuals worldwide suffer from illegal drug and alcohol usage, up from 226 million in 2010 by 21.7% [1]. Illicit drug usage cost tens of millions of disability-adjusted life years (DALYs) to the human population in 2015. The death rate was highest in low- and middle-income countries (LMICs) where data was more scarce because of an underestimation of illicit drug usage, yet Europeans suffered proportionately more [2]. Sub-Saharan Africa (SSA) is expected to have a 2.5 times higher DALYs rate of illicit drug usage than other regions [3]. The use of alcohol and illegal drugs poses a serious risk to the economy, health, and educational system [1, 2, 4, 5], contributing to the global 5.3% mortality and 5.1% morbidity. 450,000 persons died in 2015 from drug-related causes; drug use disorders directly contributed to 37.3% of these fatalities [4, 6]. According to a UNODC report, demographic surveys indicate that by 2030, there will be 11% more persons using illicit drugs globally and 40% more in Sub-Saharan Africa (SSA) due to the region’s rapidly expanding and youthful population [1]. Globally, peer pressure, low education, psychological issues, male gender, and rising rates of poverty are among the characteristics that predict adolescent drug use [7–9]. The use of illicit drugs and alcohol increases the risk of sickness, physical and mental impairment, and crime [1, 5, 10]. However, the effect on adolescents whose bodies and minds are still developing may be greater than in adults. The World Health Organization (WHO) defines adolescents as people aged 10 to 19 years [11]. One-fifth of the world’s population, or 1.2 billion adolescents, are believed to exist worldwide [3]. General demographic surveys reveal that young individuals continue to use drugs to a greater level than older persons. According to the majority of studies, early (12–14 years) to mid (15–17 years), is a critical risk period for the initiation of substance use and the onset of substance use is most likely to occur during this phase [6] because this is the time when people’s neural circuits, or brain pathways, that allow them to feel motivated and have rewarding experiences, are still active and growing [12] and regarding the parts of the brain that react mainly to new stimuli, the teenage and adult brains seem to be different. The amygdala, a brain structure located in the medial temporal lobe anterior to the hippocampus, plays a major role in how the adolescent brain reacts to unfamiliar stimuli [13]. This brain’s limbic system component manages emotions, and unpleasant emotions are linked to an increase in amygdala activation [14]. The use of substances like alcohol and illicit drugs rises in late (18–19 years old) and among young people aged 20–24 years old [6] As young people grow up the use of Illicit drugs and alcohol escalates [10, 15] and health habits acquired in adolescence including abusing alcohol or drugs are associated with grave repercussions that progress to adulthood and can last a lifetime [16, 17]. Current alcohol use among 15-17-year-olds in SSA is approximated at 21.4% by the WHO 2018 Global Status Report on Alcohol and Health [4] Furthermore, according to a systematic evaluation of research conducted in the SSA, 41.6% of adolescents reported using drugs or alcohol at any point in their lives [18]. According to reports, several important factors raise the threat of drug and alcohol abuse among teenagers in SSA, related to business sex, the availability of nonrefundable income, poverty, gender variations, and poor co-existing work and surroundings [19, 20]. Complications of illicit drug and alcohol use among adolescents are due to escalated unsafe practices. These habits increase the possibility of HIV infection and dependent syndrome which normally results in mental incapacitation [10, 19]. Adolescents in SSA are more likely to use illicit drugs and alcohol because of a variety of family, economic, and social reasons, such as extended family separation and neglect, insecure housing, the presence of drugs nearby, and moods of isolation [9, 10, 15]. While several interventions have been proposed to lower drug use in SSA, there is insufficient evidence to support their effectiveness [21, 22] Despite the established risks associated with drug use, its use continues, often even rising in response to efforts. For instance, the proportion of teenagers who believe cannabis is hazardous has decreased by 40%, despite data connecting frequent use to health issues, especially among youth [1]. In Uganda, the rates of illicit drug and alcohol use were greater among youths living in slums, people of school age, men and women fishing, and young women having high-risk sexual relationships [9, 20, 23, 24]. The population of non-school slum-dwelling adolescents and young girls and women involved in dangerous sexual behaviors remains under study [20]. Two-thirds of school-age adolescents in northern and central Uganda reported using drugs at some point in their lives, according to research [24]. Prior research conducted in Uganda’s fishing villages and capital city of Kampala has demonstrated a link between teenage drug and alcohol abuse and risky sexual practices such as frequenting paid partners and using condoms irregularly [9, 20]. The accessibility of inexpensive drugs, alcohol, and alcohol-branded products to teenagers has increased as a result of unrestricted marketing and the low cost of these substances [20, 23, 25]. However, few studies have described illicit drug and high-risk alcohol use among adolescents, and data are limited among adolescents living in urban slums where the use of these substances may be more prevalent.

In this study among male and female adolescents from urban slums in Kampala, we assessed the prevalence and baseline factors associated with illicit drug and high-risk alcohol use.

## Methodology

### Study design and setting

We conducted a cross-sectional study using data from a cohort that recruited adolescents. The main aim of the adolescent cohort was to assess the *“****F****easibility of****E****nrolling and****R****etaining****A****dolescents****at R****isk of HIV infection”* (FERDAR). Participants were recruited at the Good Health for Women Project (GHWP) clinic which was established in a peri-urban community in southern Kampala in 2008 to study the epidemiology of HIV and sexually transmitted infections (STIs) and to implement HIV/STI prevention among high-risk women with a focus on female sex workers. The GHWP clinic offered comprehensive HIV prevention, care, and treatment services to women, their children below 5 years, and regular male partners.

### Study population and recruitment

FERDAR cohort recruitment was done from 25 March 2019 to 30 March 2020 through a project field worker who mobilized 14-19-year-old female and male adolescents from six urban slums in and around Kampala. Sex work, alcohol-selling venues, and illicit drug consumption characterized these venues. Community awareness sessions were held to inform communities and adolescents about the project’s research activities. Project field workers with support from community mobilizers initially identified adolescents for screening and enrolment. Participants enrolled in the study also mobilized peers using a snowball approach.

### Eligibility criteria

Adolescents who were included in the study were 14–19 years old (consenting adolescents 18–19 years old, emancipated, and mature minors if < 18 years). Participants < 18 years met the criteria as being mature or emancipated minors. Mature Minors are individuals 14–17 years of age who have a drug or alcohol dependency or have a sexually transmitted infection. Emancipated minors are individuals below the age of maturity (18 Years), who are pregnant, married, have a child, or cater for their livelihood, as per guidelines of the Uganda National Council for Science and Technology (UNCST) [26], sexually active in the past three months, and willing to return to the clinic every 3 months for study procedures. They were excluded if they were not willing to have quarterly HIV counseling and testing provided at the clinic or intended to move away from the study area within the next 12 months.

### Enrolment

Enrolment procedures included HIV counseling and testing), participants who tested HIV positive were enrolled in the study and linked to a test and treatment program at GHWP for anti-retroviral therapy, screening, and treatment for sexually transmitted infections (STIs), a genital swab for STI testing (chlamydia, gonorrhea) and a syphilis sample was collected. Participants found positive for STIs were given treatment, and their partners were invited for testing and treatment. Contraceptive refills and pregnancy tests were done for female adolescents and those found pregnant were enrolled and linked to ante-natal care services.

Illicit drug and high-risk alcohol use counseling was offered to enrolled participants.

### Counselling for high-risk alcohol and illicit drug use

Simple advice on reduction of low-risk and hazardous drinking (1–7 and 8–15 AUDIT scores, respectively) was given, brief counseling and continued monitoring was provided for high-risk/harmful alcohol use (16–19 AUDIT scores), diagnostic evaluation for alcohol dependence (≥ 20 AUDIT scores) and referral was recommended as in the WHO AUDIT tool guidelines for use in primary care, second edition [27, 28].

### Laboratory procedures

HIV testing was done on serum using the Ministry of Health (MoH) algorithm (Determine, Statpak, and SD Bioline). Statpak confirmed positive Determine and SD Bioline were used as tie-breakers. For serum syphilis testing, we used the Rapid Plasma Reagin (RPR) for screening and treponema pallidum particle agglutination assay (TPPA) as a confirmatory test. A genital swab was taken for STI testing (chlamydia, gonorrhea) using Roche Cobas x4800 Real-time polymerase chain reaction. A pregnancy test was done on urine, Human Chorionic Gonadotrophin Hormone using a pal urine dipstick strip.

### Data Collection

Trained research assistants collected data using interviewer-administered questionnaires that were translated into the local language of the area (Luganda). Both English and Luganda version of the questionnaires were available depending on the participant’s language of choice. We collected data on socio-demographic, sexual behavior, and reproductive health variables, illicit drug use at enrolment and in the past three months using the social-demographic risk behavior questionnaire, and alcohol use at enrolment using the AUDIT questionnaire. This standard tool captures alcohol use in the past 12 months.

### Study variables

#### Dependent (outcome) variables included

- Illicit drug use in the past 3 months at enrolment and this was measured as a binary variable (Yes/No). Participants were asked the following question: In the last 3 months, did you use any drugs of addiction (1 = Yes, 2 = No).
- High-risk alcohol consumption at enrolment was measured as a binary variable. This was based on the score of the Alcohol Use Disorders Identification Test (AUDIT) questionnaire which was developed by WHO [29] The test assesses the use of alcohol during the previous 12 months and consists of 10 screening questions relating to alcohol consumption; drinking behavior and dependence; and problems associated with alcohol. Scores for all 10 questions were summed up and categorized as follows: 0–7 (low-risk drinking/abstinence), 8–15 (moderate-risk/hazardous drinking), 16–19 (high-risk/harmful drinking), and ≥ 20 (high-risk/alcohol dependent). This was further categorized into a binary variable; high risk (AUDIT score ≥ 16) vs. low to moderate risk (0–15) [28].

#### Independent variables included

- Socio-demographic variables included the participant’s current age, age at first sexual intercourse, average monthly income, marital status, gender, level of education, and sexual and reproductive health variables; the latter included the total number of sexual partners at enrolment and as well as in the past three months, number of biological children, number of abortions and miscarriages ever experienced, the experience of intimate partner violence (IPV) from sexual partners in the past 3 months (yes/no), engaging in paid sex in the past 3 months (yes/no), condom use with partners at the last sexual encounter (yes/no), type of sexual partner (regular partner living together, regular partners not living together, paying partners, casual acquaintance or any other partners), and contraceptive use for females (yes/ no).

### Statistical analysis

- Data were double-entered in Open Clinica, cleaned, and exported to Stata17.0 (Stata Corp, College Station, TX, USA) for further management and analysis. The participants’ characteristics were summarized in descriptive terms such as mean, median, standard deviations, or percentage, as appropriate. The proportions of illicit drug use and high-risk alcohol use at baseline were calculated as proportions of the total number enrolled. Factors associated with illicit drug use at baseline and in the past 3 months and high-risk alcohol use at baseline were determined for each outcome and each independent variable using logistic regression. Bivariate/unadjusted logistic regression analysis was conducted and variables with *p*-value < 0.15 were considered for the adjusted, multivariable analysis. Multivariable logistic regression was used to identify the factors that are independently associated with illicit drugs and high-risk alcohol use. Each outcome was considered separately, with its model. In the multivariable analysis, variables were kept in the model if removing them significantly affected model fit. Variables that had a *P*-value < 0.05 were considered considerably associated with illicit drug and high-risk alcohol use. In both models, we considered age at enrolment and sex as priori confounders included in the multivariable models regardless of their unadjusted *p*-values.

## Results

### Baseline characteristics of enrolled participants

- We included 490 participants in our analysis; 297 (60.6%) females and 193 (39.4%) males. The median age of participants was 18 (IQR 17–18) years, 249(50.8%) were ≥ 18 years, and 416(84.9%) had less than secondary education. Of 237 (48.4%) adolescents who had their sexual debut before 15 years, 134 (56.5%) were female vs. 103(43.5%) males. Participants who had any STI (chlamydia, gonorrhea, and active syphilis) at enrollment were 111(22.8%), of whom 75.7(84.7%) were female. Of 231 (47.1%) who reported paid sex in the past 3 months, 219(94.4%) were female. Among the 219 females who reported paid sex in the past 3 months, 33(75.0%) had a higher than secondary level of education. Adolescents reporting more than one sexual partner were 332 (67.8%) of whom 62.9% were female. Female adolescents using contraception were 158 (59.2%). Also of the 154 (31.4%) who had biological children 140(90.9%) were females. (Table 1).

**Table 1:** Baseline characteristics of adolescents enrolled from Kampala slums, Uganda

Variable	Category	Males *n* (row%)	Females *n* (row%)	Total *n* (col%)
**Overall**		193(39.4)	297(60.6)	490(100)
Age at enrollment				
	14-17years	89(36.9)	152(63.1)	241(49.2)
	≥ 18 years	104(41.8)	145(58.2)	249(50.8)
Education level				
	None	10(33.3)	20(66.7)	30(6.1)
	Less than secondary	172(41.4)	244(58.6)	416(84.9)
	Secondary/higher	11(25.0)	33(75.0)	44(8.9)
Age-first sexual intercourse				
	< 15 years	103(43.5)	134(56.5)	237(48.4)
	≥ 15 years	90(35.6)	163(64.4)	253(51.6)
Marital status				
	Never married	174(46.1)	203(53.9)	377(77.1)
	Currently married	13(20.0)	52(80.0)	65(13.3)
	Separated/ /Divorced	6(12.5)	42(87.5)	48(9.6)
Have biological children				
	Yes	14(9.1)	140(90.9)	154(31.4)
	No	179(53.3)	157(46.7)	336(68.6)
Monthly Income ($).				
	None	4(6.4)	59(93.6)	63(12.9)
	< 30$	76(36.7)	131(63.3)	207(42.2)
	> 30$	113 (51.4)	107(48.6)	220(44.9)
Number of sexual partners in the past three months				
	1	70(44.3)	88(55.7)	158(32.2)
	≥ 2	123(37.1)	209(62.9)	332(67.8)
Paid sex in the past 3 months				
	Yes	12(5.2)	219(94.8)	231(47.1)
	No	181(69.9)	78(30.1)	259(52.9)
Current use of illicit drugs				
	Yes	119(69.6)	52(30.4)	171(34.9)
	No	74(23.2)	245(76.8)	319(65.1)
High-risk alcohol drinking				
	Yes	21(26.6)	58(73.4)	79(16.1)
	No	172(41.9)	239(58.1)	411(83.9)
On contraception*				
	Yes		158(100.0)	158(59.2)
	No		109(100.0)	109(40.8)
Ever tested for HIV				
	Yes	130(33.2)	262(66.8)	392(80.0)
	No	63(64.3)	35(35.7)	98(20.0)
Any STI (chlamydia, gonorrhea, and/or active syphilis)^z^				
	Yes	27(24.3)	84(75.7)	111(22.8)
	No	166(44.2)	210(55.9)	376(77.2)

* Only females, defined as reporting contraception (3-monthly Depo-Provera injection, Intra Uterine Contraceptive Device (IUCD), Combined oral contraceptives and implant.)

^***z***^ ***N= 487, excludes 3 participants not tested for STIs***

### Illicit drug and high-risk alcohol use among adolescents

**Fig. 1:**
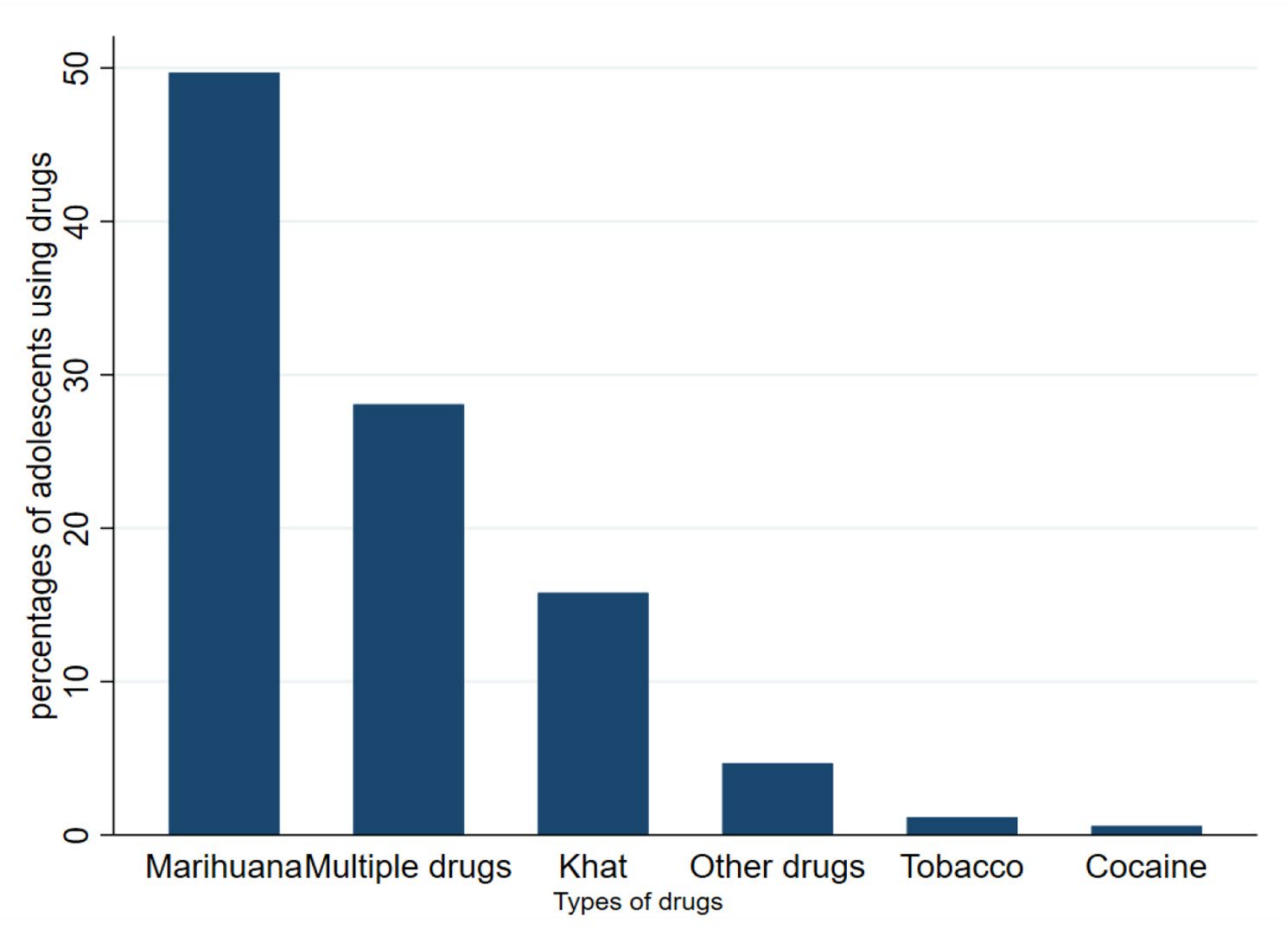
Percentages of the types of illicit drugs used by adolescents

At enrollment, 224 participants had ever used illicit drugs of whom 141 (62.9%) were male. Current Illicit drug users at enrolment (past 3 months) were 171 (34.9%) with the proportion of those using Illicit drugs among males significantly higher than females (69.6% vs. 30.4%, *p* < 0.001), and 86 participants (50.3%) of current drug users reported using drugs daily (76.7% male’s vs. 23.3% females), 68 (39.7%) reported using drugs once a week (64.7% males’ vs. 35.3% females) while the rest used drugs less than once a month. The most commonly used drugs for both males and females were marijuana 85 (49.7%) and Khat 27(15.8%) (Fig. 1). Reasons given by males for taking illicit drugs were: to forget their problems 38 (31.9%), to feel good 29(24.4%), to peer pressure 14(11.8%), to get courage so they could do their work 4(3.4%), and other reasons 34(28.6%). Reasons given by females were peer pressure 15(28.9%), to feel good 12(23.1%), to get the courage to engage in sex work 10(19.2%), and other reasons 15(28.9%).

### Baseline characteristics associated with illicit drugs

At adjusted analysis, baseline characteristics associated with illicit drug use in the past 3 months were male gender (aOR 12.45; 95% CI 7.21–21.50) compared to females, being married (aOR 2.26; 95%CI 1.03–4.94) compared to being single, having 10 or more paying sexual partners (aOR 2.45; 95%CI 1.05–5.69) compared to reporting fewer than 10 and high-risk alcohol users compared to the non-high-risk alcohol users (aOR 3.94; 95%CI 2.10–7.40) (Table 2).

**Table 2:** Association of participants’ baseline characteristics with illicit drug use in the past 3 months

Baseline Characteristics	Overall *N* = 490 *n* (%col)	Used illicit drugs ( *n* = 171) *n*( row%)	Unadjusted OR (95% CI)	*P*-value	Adjusted* OR (95% CI)	*P*-value
**Age at enrolment**						
14–17	241(49.2)	82(34.0)	1(ref)		1(ref)	
18–19	249(50.8)	89(35.7)	1.08(0.74–1.56)	0.69	1.02(0.65–1.60)	0.925
**Sex**						
Female	297(60.6)	52(17.5)	1(ref)		1(ref)	
Male	193(39.4)	119(61.7)	7.58(4.99–11.49)	< 0.001	**12.45(7.21–21.50)**	**< 0.001**
**Cater for own livelihood**						
No	82(16.7)	15(18.3)	1(ref)		1(ref)	
Yes	408(83.3)	156(38.2)	2.77(1.53–5.01)	0.001	1.50(0.70–3.22)	0.296
**Marital status.**						
Single	377(76.9)	135(35.8)	1(ref)		1(ref)	
Separated/divorced	48(9.8)	15(31.2)	0.81(0.43–1.55)	0.534	1.91(0.87–4.20)	0.105
Married	65(13.3)	21(32.3)	0.86(0.49–1.49)	0.586	**2.26(1.03–4.94)**	**0.040**
**Ever been to school**						
Yes	460(93.9)	159(34.6)	1(ref)			
No	30(6.1)	12(40.0)	1.26(0.59–2.69)	0.546		
**Education level**						
Secondary or higher	44(9.0)	9(20.5)	1(ref)			
Less than secondary.	446(91.0)	162(36.3)	2.22(1.04–4.73)	0.039	1.55(0.63–3.82)	0.331
**Age at first Job**						
First job < 15yrs	187(38.2)	80(43.0)	1(ref)			
First job at > = 15yrs	303(61.8)	91(30.0)	0.52(0.35–1.76)	0.001	0.74(0.47–1.17)	0.202
**Main job**						
Sex work	52(10.6)	17(32.7)	1(ref)			
No job	60(12.2)	11(18.3)	0.46(0.19–1.11)	0.083		
Other jobs	378(77.1)	143(37.8)	1.25(0.68–2.32)	0.473		
**Paying sexual partners in the last 3 months**						
< 10 partners	453(92.4)	158(34.9)	1(ref)		1 (ref)	
>=10 partners	37(7.6)	13(35.1)	1.01(0.50–2.04)	0.975	**2.45(1.05–5.69)**	**0.036**
**Reported condom use with paying partners in the last 3 months**						
Inconsistent	154(66.1)	36(23.4)	1(ref)			
Consistent	79(33.9)	19(24,0	1.03(0.54–1.96)	0.909		
**Experienced physical violence from sexual partners in the past 3 months**						
No	427(87.1)	148 [30]	1(ref)			
Yes	63(12.9)	23 [31]	1.08(0.63–1.88)	0.774		
**Ever experienced sexual violence from sexual partners in the past 3 months**						
No	401(81.8)	157 [30]	1(ref)			
Yes	89(18.2)	14 [31]	1.09(0.55–2.18)	0.794		
**Ever experienced psychological /emotional Violence from sexual partners in the past 3 months**						
No	403(74.9)	138 [32]	1(ref)			
Yes	87(25.1)	33 [33]	1.17(0.73–1.89)	0.513		
**Alcohol Use**						
No	411(83.9)	130(31.6)	1		1	
Yes	79(16.1)	41(51.9)	2.33(1.43–3.79)	0.001	**4.27(2.34–7.81)**	**< 0.001**

*adjusted for age, sex, marital status, attending school, first job, whether one caters for their livelihood, paid sex at last sexual encounter, and violence from sexual partners; OR = odds ratio

### Baseline characteristics associated with high-risk alcohol use

At adjusted analysis, baseline characteristics associated with high-risk alcohol use were male gender (aOR 0.29; 95% CI 0.13–0.63) compared to females, having experienced emotional violence from sexual partners (aOR 2.35; 95%CI 1.32–418) compared to those who did not report IPV and illicit drug users compared to the non-drug users (aOR 3.94; 95% CI 2.10–7.40) (Table 3).

**Table 3:** Association of participants’ baseline characteristics with high-risk alcohol use

Baseline Characteristics	Overall *N* = 490 *n* (col%)	High risk alcohol use (*n* = 79) *n*( row%)	Unadjusted OR (95% CI)	*P*-value	Adjusted* OR (95% CI)	*P*-value
**Age at enrolment**						
14–17	241(49.2)	37(15.3)	1(ref)		1(ref)	
18–19	249(50.8)	42(16.9)	1.12(0.69–1.81)	0.649	1.35(0.79–2.30)	0.267
**Sex**						
Female	297(60.6)	58(19.5)	1(ref)		1(ref)	
Male	193(39.4)	21(10.8)	0.50(0.29–0.86)	0.012	**0.29(0.13–0.63)**	**0.002**
**Cater for own livelihood**						
Yes	82(16.7)	65(15.9)	1(ref)			
No	408(83.3)	14(17.0)	1.09(0.58–2.05)	0.798		
**Marital status**						
Single	377(76.9)	55(14.6)	1(ref)		1	
Separated/divorced	48(9.8)	9(18.8)	1.35(0.62–2.94)	0.449	0.59 (0.23–1.53)	0.282
Married	65(13.3)	15(23.0)	1.76(0.92–3.34)	0.086	1.50(0.73–3.08)	0.268
**Ever been to school**						
Yes	460(93.9)	72(15.6)	1(ref)			
No	30(6.1)	7(23.3)	1.64(0.68–3.96)	0.272		
**Education level**						
Less than secondary	44(9.0)	72(16.1)	1(ref)			
Secondary or higher	446(91.0)	7(15.9)	0.98(0.42–2.29)	0.968		
**Age at first Job**						
First job at < 15yrs	187(38.2)	33(18.0)	1(ref)			
First job at > = 15yrs	303(61.8)	46(15.1)	0.82(0.50–1.36)	0.452		
**Main job**						
No job	52(10.6)	6(10.0)	1(ref)		1(ref)	
Sex work	60(12.2)	17(32.7)	4.37(1.57–12.16)	0.005	2.93 (0.91–9.45)	0.071
Other Jobs	378(77.1)	56(14.8)	1.57(0.64–3.81)	0.324	2.20(0.83–5.82)	0.111
**I have been paying sexual partners for the last 3 months**.						
< 10 partners	453(92.4)	66(14.6)	1(ref)		1	
>=10 partners	37(7.6)	13(35.1)	3.18(1.54–6.55)	0.002	1.80 (0.70–4.59)	0.216
**They reported condom use with paying partners in the last 3 months.**						
Inconsistent	154(66.1)	40(25.9)	1(ref)			
Consistent	79(33.9)	16(20.2)	0.72(0.38–1.39)	0.334		
**Experienced physical violence from sexual partners in the past 3 months**						
No	427(87.1)	60(14.0)	1(ref)		1(ref)	
Yes	63(12.9)	19(30.1)	2.64(1.44–4.83)	0.002	1.75(0.87–3.51)	0.112
**Ever experienced sexual violence from sexual partners in the past 3 months**						
No	401(81.8)	65 [15]	1(ref)		1	
Yes	89(18.2)	14 [34]	3.47(1.70–7.06)	0.001	1.78(0.77–4.12)	0.173
**Ever experienced psychological /emotional Violence from sexual partners in the past 3 months**						
No	403(82.2)	53(13.1)	1(ref)		1(ref)	
Yes	87(17.8)	26(29.8)	2.81(1.64–4.84)	< 0.001	**2.14(1.14–4.12)**	**0.018**
**Drug use**						
No	319(65.1)	38(11.9)	1		**1**	
Yes	171(34.9)	41(23.9)	2.33(1.43–3.79)	0.001	**3.94 (2.10–7.40)**	**< 0.001**

*adjusted for age, sex, attending school, whether one caters for their livelihood, paid sex at last sexual encounter; OR = Adjusted odds ratio

## Discussion

Our study found a high prevalence of illicit drug and high-risk alcohol use among 14-19-year-old adolescents. This is perhaps not surprising, given that the source GHWP clinic provides services to participants from Kampala slums that are characterized by a high prevalence of crime, and illicit drug and alcohol use. This environment exposed adolescents to the use of these substances and this is consistent with other research conducted on vulnerable teenagers, adolescent girls, and young women living in Kampala Slums and Sub-Saharan Africa that portray a high prevalence of illicit drug and alcohol use [18, 23, 35]. However, this differs from a study done by Berhane et al. in nine communities in SSA that reported a very low prevalence of alcohol and illicit drug use among both school and non-school-going adolescents 10–19 years [16].

Illicit drug and alcohol use in our study was associated with being male, male participants in our cohort were married young, some had more than 10 paying sexual partners in the past three months, and some experienced Intimate Partner Violence from their sexual partners, they, therefore, reported resorting to the use of drugs and alcohol to forget their problems caused by adolescent marriage, sexual partners’ experiences with IPV [36, 37], and to feel good [24, 38]. Women in East Africa, SSA, and some parts of the world are discriminated against because they are using drugs and are expected to act and behave a certain way by culture and society [30, 32], They are professed as unsuitable, outrageous, and humiliating which is not the case with the males. This may be linked to higher rates of substance and alcohol use among males compared to females in Uganda and SSA, this is in agreement with a Tanzanian study that portrayed lower odds of illicit drug use among female adolescents compared to males due to social and cultural conventions about women and men using drugs [32] higher odds of males using alcohol is in line with a study done in Ibadan Nigeria which found being male significant to alcohol use [25]. We further found that marijuana and khat were the most used drugs by adolescents and when compared to other medications, both of them are more accessible and economical for adolescents with modest incomes because they are readily available locally and are inexpensive to buy. This is similar to studies done in Uganda and East Africa among adolescents in school and fishing communities [9, 32]. More than half of male participants who used drugs reported using them as a coping mechanism to forget their problems and to feel good and content [24, 38].

Another significant factor of the current study, illicit drug use was associated with being married, attributed to the fact that most of the married participants in this study got married young, and a high percentage had less than secondary education, they, therefore, declared having experienced intimate partner violence (IPV) in their marriage relationships, lacking formal and parental supervision and direction. They stayed alone or with friends and indulged in early marriages [34, 36, 37]. This is however contrary to the first part of the study analysis done in Washington which discovered that partner use has a significant role in moderating the relationships between excessive drinking and marijuana use [31], our study results are in line with the relationship quality of the same study, regardless of the status of the relationships. Less usage was only associated with more fulfilling, gratifying, and supportive relationships when the intimate partner did not engage in significant substance use and IPV. These results demonstrate the impact of relationship attachment, partnering behavior acceptance, and the interplay between these two processes on adolescent substance use [31].

This study confirms earlier findings that demonstrate a high correlation between drug and alcohol use [17, 25, 33]. It seems that in adolescence, unhealthy habits lead to additional harmful behaviors. The odds of an adolescent using alcohol were significant to illicit drug use [17] and the odds of one engaging in high-risk alcohol use were higher when using Illicit drug use [33]. This is in line with studies done among adolescents and female sex workers in SSA, Nigeria, Kenya, and Uganda that report a high association between illicit drug and alcohol use [23, 31, 39].

The current research also indicated that having 10 or more paying partners was associated with illicit drug use. Almost three-quarters of our enrolled females reported engaging in paid sexual intercourse with multiple sexual partners at enrolment and in the past 3 months, most adolescents had low or no education. This holds significance to the components of adolescent health where numerous research indicate that drug usage and educational qualification are positively associated as described by Arasi. O and colleagues in a study done in Nigeria [25]. Most adolescents worked in bars as sex workers to earn a living where they usually met their sexual customers who would buy them alcohol and drugs before engaging in sexual activities [19, 25, 40]. Some participants in this study started sex work as their first job, they reported using drugs and alcohol to be bold and confident while doing their job [23], and to deal with the stress of being a sex worker and a drug user, which includes prejudice and imprisonment in Uganda [40, 41] Having started sex work at an early age as a main job, adolescents in this study started using alcohol at an early age as well and Long-term alcohol use eventually led to high-risk alcohol use influenced by the duration of sex work, our results are comparable to results from a study done among adolescent girls and young women involved in high-risk sexual behavior in Kampala by Mayanja Y and colleagues’ providing evidence that sex work as a main job predispose young girls and women to high-risk alcohol use [23, 40]. Adolescents reported using alcohol because their friends were using, these teenagers’ participation in related high-risk activities, such as having many sexual partners, and their age groups that were equivalent to alcohol use were impacted by their peers although peer pressure was not significant in our study other studies in SSA and Uganda have reported peer pressure being influential in prevalent drug and alcohol use, a study done among Zambian street youth who have similar characteristics as adolescents in this study, has confirmed teens living in urban slums being less likely to use illicit drugs and alcohol if they have a far better understanding of their friends’ behavior who are not using [41].

We further found that adolescents who experienced physical and emotional violence from sexual partners in this study were more likely to be high-risk alcohol users [23, 42]. This is because most of our study participants engaged in paid sex and because they were young and vulnerable, they lacked strong negotiating positions, and they were exposed to Intimate Partner Violence from their sexual prospects especially when they were high on alcohol [43, 44], they therefore continued to use alcohol to cope with violence from sexual partners like how Monica. H. Shahn and colleagues’ findings were among girls and young women living in Kampala slums, most of whom were in the same age range as adolescents in this study. There is an ambiguous connection between reporting multiple kinds of rage and drunkenness [39].

## Strength, limitations, and recommendations

A random selection was used to create the study sample to discover and mobilize volunteers by using community mobilizers, adolescent peers, and health field workers, a study strength however, it is uncertain how this selection may affect the findings.

Longitudinal studies are required to explain patterns and trends of illicit drug and alcohol use among adolescents living in Kampala slums to describe the extent of damage versus mitigating factors to drug and alcohol use.

In order to create a safe and fulfilling drug and alcohol-beginning life for teenagers, the early years of adolescence are essential. Comparing outcomes and variables between early and late adolescence requires research that splits the adolescent years into two categories: early [10–14] and late [15–19].

Given that participants were to be followed up 3-monthly for a year in the main study a longitudinal study design would have yielded more significant results to explain and understand the patterns and trends of illicit drug and alcohol use among slum-dwelling adolescents compared to the cross-sectional study design we conducted preventing our ability to infer causality in the associations between dependent and independent outcomes. Our study was done among a unique population of adolescents that also included emancipated minors. Since only 14–19-year-olds were included in the study, leaving out 10–13-year-old young adolescents, the study’s findings may underestimate the full burden of adolescents living in slums and results may not be generalizable to all adolescents in different settings and those who need parental/ guardian consent.

## Conclusion

Illicit drug and high-risk alcohol use were prevalent among adolescents from urban slums in Kampala. Male adolescents and those who report vulnerabilities like IPV, multiple sexual partnerships, and sex work have a higher prevalence of substance use. Slum life is a dangerous setting with unacceptable risky and unhealthy living circumstances and it is critical to comprehend the larger socio-ecological background of these slums to enhance and advance the overall health of teenagers living in slums.

Comprehensive interventions such as adolescent drug and alcohol use counseling, integrated adolescent care package which includes sexual reproductive health and rights, substance use and mental health among others, adolescent involvement in health care implementation, strict Laws on drugs and alcohol use among adolescents, family and community modeling, and construction of more rehabilitation centers are needed by adolescents living in urban slums to reduce illicit drug and alcohol use to prevent negative long-term health consequences that may persist into adulthood. and should include measures against intimate partner violence.

## Data Availability

The datasets used and analyzed during the current study are available from the corresponding author.
